# Mitochondrial-associated metabolic disorders: foundations, pathologies and recent progress

**DOI:** 10.1186/1743-7075-10-63

**Published:** 2013-10-12

**Authors:** Joseph McInnes

**Affiliations:** 1 School of Engineering and Science, Research Center MOLIFE – Molecular Life Science, Jacobs University Bremen, Campus Ring 1, Research II, Room 120, Bremen D-28759, Germany; 2 VIB Center for Biology of Disease, Katholieke Universiteit Leuven, Leuven, Belgium; 3 Center for Human Genetics and Leuven Research Institute for Neurodegenerative Diseases (LIND), Katholieke Universiteit Leuven, Leuven, Belgium

**Keywords:** Mitochondria, mtDNA, Metabolism, Disease

## Abstract

Research in the last decade has revolutionized the way in which we view mitochondria. Mitochondria are no longer viewed solely as cellular powerhouses; rather, mitochondria are now understood to be vibrant, mobile structures, constantly undergoing fusion and fission, and engaging in intimate interactions with other cellular compartments and structures. Findings have implicated mitochondria in a wide variety of cellular processes and molecular interactions, such as calcium buffering, lipid flux, and intracellular signaling. As such, it does not come as a surprise that an increasing number of human pathologies have been associated with functional defects in mitochondria. The difficulty in understanding and treating human pathologies caused by mitochondrial dysfunction arises from the complex relationships between mitochondria and other cellular processes, as well as the genetic background of such diseases. This review attempts to provide a summary of the background knowledge and recent developments in mitochondrial processes relating to mitochondrial-associated metabolic diseases arising from defects or deficiencies in mitochondrial function, as well as insights into current and future avenues for investigation.

## Introduction to mitochondrial processes

### General roles of mitochondria in metabolism

In its most traditional definition, the mitochondrion is the energy-generating organelle of the cell, responsible for the final steps of metabolizing organic substances to produce energy for the cell in the form of adenosine triphosphate (ATP). In mammalian cells, most of the redox potential used for generating ATP arrives at the mitochondrion in the form of the nicotinamide adenine dinucleotide (NADH) and flavin adenine dinucleotide (FADH_2_), reduced coenzymes generated by the acceptance of electrons derived from the breakdown of organic substances in the tricarboxylic acid (TCA) cycle. Four protein complexes in the inner membrane make up the electron transport chain (ETC, also known as the electron transport system), which converts the redox energy stored as NADH and FADH_2_ into chemical energy in the form of ATP. In brief, complexes I and II receive electrons donated from NADH and FADH_2_, respectively, followed by their shuttling to complexes III and IV, and their final donation onto an oxygen molecule, yielding H_2_O. Additional proteins can transfer electrons from donors to the ETC, including electron-transferring-flavoprotein dehydrogenase (member of a pathway receiving electrons from β-oxidation and amino acid catabolism) [1], and CHCHD4, which receives electrons from disulfide bridges [2]. As electrons are shuttled through the complexes I, III and IV by electron carriers, protons (H^+^) are moved from the mitochondrial matrix into the intermembrane space. This creates a net positive charge in the intermembrane space, and a net negative charge in the matrix. This electrochemical gradient is responsible for providing the driving force needed to produce ATP [3].

F_1_F_0_-ATP synthase is a large enzyme complex made up of over 22 subunits. The preferred route for protons in the intermembrane space to re-enter the matrix, in an attempt to flow down the gradient and re-establish equilibrium, is to enter through the ATP synthase enzyme complex. As protons flow through the channel domain of ATP synthase, a motor force is generated, which is used to rotate a large, rotating catalytic domain facing the matrix, which couples adenosine diphosphate (ADP) to an inorganic phosphate moiety (P_i_) to yield ATP [4]. Experiments using purified enzymes from cows and yeast revealed that the number of protons required by ATP synthase to make a single ATP molecule is almost twice as high for lower eukaryotes as it is for animals [5]. Thus, the production of ATP by the ATP synthase enzyme has significantly increased in efficiency during the evolution of multicellular organisms from early eukaryotic ancestors.

Mitochondria most readily produce ATP by the oxidation of NADH and FADH_2_ yielded from the breakdown of sugars such as glucose. In mammals, however, a second energy source comes from the degradation of fatty acids, some of which are liberated from lipid reserves, for example in adipocytes. Fatty acids, which are often found in chains as CoA-esters, are first transported across the outer mitochondrial membrane by palmitoyl transferase I, resulting in translocation to the intermembrane space and transformation into an acyl-carnitine form. Subsequently, acyl-carnitines are translocated across the inner mitochondrial membrane by cartinine acyl translocase. Once in the matrix, acyl-carnitines are converted back to their original acyl-CoA form by palmitoyl transferase II, which can be oxidized to yield energy [6]. β-Oxidation is catalyzed by four matrix-localized enzymes, and results in the acyl-CoA entering the citric acid cycle to yield NADH and FADH_2_, which are utilized by the ETC for ATP production [7]. In humans, β-oxidation is a major energy source, and defects in this process, or in the ETC in general, can lead to ATP deficiency, as explored further below (Table 1).

**Table 1:** Common mutations in genes or gene groups resulting in mitochondrial-associated metabolic diseases

**Gene(s)**	**Location**	**Product**	**Function**	**Phenotype**	**Associated diseases**
*COQ2, COQ9, PDSS1/2, ADCK3*	Nuclear	Coenzyme Q_10_ (CoQ)	Required for synthesis of CoQ; CoQ is an essential electron shuttle, acts as antioxidant, cofactor of pyrimidine synthesis	Decreased ETC activity; Skeletal muscle breakdown	CoQ deficiency
ETC subunits	Mitochondrial	Mitochondrial respiratory complex subunits	Oxidative phosphorylation	Respiratory complex deficiency	Mitochondrial myopathy; Lactic acidosis; MELAS (complex I)
*ETFDH*	Nuclear	Electron-transferring-flavoprotein dehydrogenase	Required for delivering electrons to complex III; derived from fatty acid and amino acid oxidation	Accumulation of toxic intermediates from fatty acid and amino acid oxidation; Decreased ETC efficiency	Symptoms of glutaric acidemia type 2
*POLG, POLG2*	Nuclear	Mitochondrial DNA polymerase gamma	mtDNA replication	mtDNA depletion	mtDNA depletion syndrome; Symptoms of MNGIE
12S rRNA, 16S rRNA	Mitochondrial	Mitochondrial ribosomal RNAs	Required for synthesis of mtDNA-encoded proteins	Respiratory complex deficiency	Mitochondrial myopathy; Lactic acidosis
tRNAs	Mitochondrial	Mitochondrial transfer RNAs	Required for synthesis of mtDNA-encoded proteins	Respiratory complex deficiency	Mitochondrial myopathy; Lactic acidosis; MELAS
*TYMP*	Nuclear	Thymidine phosphorylase	Required for processing thymidine and deoxyuridine into thymine and uracil	Accumulation of thymidine and deoxyuridine; Imbalance in mitochondrial (d)NTP pool; Impaired mtDNA replication and transcription	MNGIE

### The mitochondrial life cycle: fusion, fission, and autophagy

**Figure 1:**
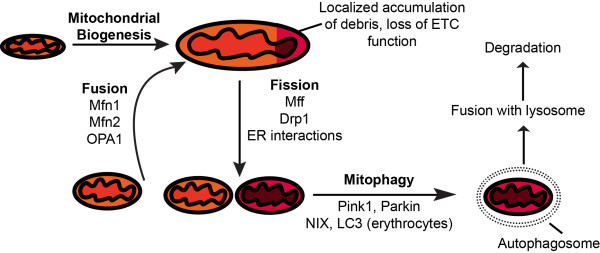
**The mitochondrial life cycle.** Since mitochondria cannot be synthesized *de novo*, they must arise from existing mitochondria. Mitochondrial biogenesis is the process by which mitochondria increase in size, accompanied by lipid synthesis and assembly of ETC subunits. One mitochondrion can divide into two physically distinct mitochondria by the process of fission, which requires the mechanical force of the ER and the Drp1 protein, which is recruited to mitochondria by the Mff receptor. Two mitochondria can also join together to become one mitochondrion with continuous inner- and outer-membranes in a process termed mitochondrial fusion, which requires the proteins Mfn1, Mfn2, and OPA1. In the case of mitochondrial damage, indicated by decreased ETC activity, oxidizing membrane potential, and accumulation of ROS and unfolded proteins, a mitochondrion may be degraded by mitophagy. The marking of mitochondria for degradation is facilitated by the NIX/LC3 pathway during erythrocyte differentiation, and by the Pink1/Parkin pathway in other cell types.

Mitochondria cannot be synthesized *de novo*, and thus new mitochondria must arise from existing mitochondria. Similar to a cell growing before division, a mitochondrion elongates and increases in volume before septation, which results in two physically separate mitochondria (Figure 1). This process is termed mitochondrial fission. Notably, mitochondria also have the ability to fuse together, joining membranes and compartments to yield a single larger mitochondrion. Mitochondrial fusion and fission occur throughout the life cycle of the cell, meaning that at any time point, mitochondria are in a dynamic flux between fusion and fission. It is believed that this balance of fusion and fission gives rise to the diverse mitochondrial sizes and structures visible when observing mitochondrial morphology [8].

Proteins essential for mitochondrial fusion have been relatively well described, including the GTPases Mfn1, Mfn2 and OPA1. The mitofusins Mfn1 and Mfn2, along with a dynamin-related GTPase OPA1, are three proteins required for mitochondrial fusion in mammals. Mitofusins localize to the outer mitochondrial membrane, where they facilitate outer membrane fusion, while OPA1 localizes to the inner membrane, where it facilitates inner membrane fusion. Mfn1 and Mfn2 are required to be on both mitochondria for fusion to occur, while OPA1 present on only one of the two mitochondria is sufficient for inner membrane fusion [9]. As the two mitochondrial objects approach each other, the inner and outer mitochondrial membranes fuse simultaneously, preventing contents of the matrix and intermembrane space from mixing, and thereby maintaining mitochondrial function.

The opposite process of mitochondrial fusion is fission, whereby one mitochondrion divides and results in two physically distinct mitochondria. In contrast to fusion, the key players in mitochondrial fission require more investigation, as the current picture is incomplete. Most recent evidence implicates the dynamin-related Drp1 protein to play the central role in mitochondrial fission, by the use of a GTPase domain to catalyze a membrane-pinching action similar to the mechanism of vesicle endocytosis [10]. Interestingly, Drp1 localizes to the cytosol, suggesting the presence of a mitochondrial membrane-bound receptor to recruit Drp1. Despite initial reports that the Fis1 protein plays the role of a Drp1 receptor [11], most recent evidence suggests the Mff protein plays this role, and has been shown to be essential for Drp1 recruitment [12]. Most recently, the picture of mitochondrial fission has become even more complex with the finding that the mitochondrial interactions with the endoplasmic reticulum (ER) plays a role in the recruitment of Drp1 to Mff receptors, and possibly provides a mechanical force to facilitate fission [13]. This finding adds to the growing list of processes that are mediated by mitochondria-ER interactions (see below).

It is known that the function of the dynamin-related GTPases involved in mitochondrial fission work similarly to those involved in other membrane-restricting processes, such as the fission of peroxisomes. However, exactly which proteins play key roles in this process, as well as how and when they are recruited and regulated, as well as their interaction at mitochondria-ER contact sites, remains a large feat for future research. Additionally, deficiencies or mutations in any of the players in mitochondrial fusion or fission are implicated in severe pathologies. For example, it has been shown that a mutation in OPA1, required for mitochondrial fusion, leads to deficits in mitochondrial respiration and ultimately death in retinal ganglion cells, resulting in blindness [14].

Mitochondrial fission is not only important for the division of mitochondria; it is also used as a mitochondrial waste disposal system, which is crucial for maintaining mitochondrial respiration. Throughout its lifetime, a mitochondrion can accumulate damage and debris, characterized by excessive amounts of reactive oxygen species (ROS) and dysfunctional proteins, often leading to uncoupled electron transport chains. One way mitochondria can dispose of such molecular debris is by segregating that part of the mitochondrion by fission, followed by its recruitment into an autophagosome for mitophagy [15].

Mechanisms for mitophagy in mammals have been highly conserved from yeast, and are believed to be a vital mechanism for preventing metabolic disease in humans [16,17]. Upon loss of membrane potential, the Pink1 protein marks mitochondria for degradation by recruiting the ubiquitin ligase Parkin, which creates ubiquitin tags on the mitochondrion, marking it for degradation and recruiting an autophagosomal membrane to the mitochondrion [18]. Eventually, the autophagosome will fuse with a lysosome, at which point its contents will be degraded and recycled. This process has been well understood in red blood cells, which utilize a similar pathway for the depletion of mitochondria during the maturation of reticulocytes into erythrocytes [19]. During erythrocyte maturation, the NIX protein attaches to mitochondria and recruits the LC3 protein, which is bound to an autophagosomal membrane to encircle the mitochondrion for degradation [16].

Finally, defects in mitophagy have been linked to human pathologies associated with metabolism such as ageing, cancer, neurodegenerative disease, and tissue injury and repair [17,20]. Additionally, mitophagy now appears to be one pathway to suppress mitochondrial DNA (mtDNA) mutations [21]. Growing evidence provides more linkages between mitophagy and a number of cellular functions and pathologies [22], highlighting the importance of further investigating mitophagy in the context of metabolic disease.

### Maintenance of mtDNA is critical for metabolic function

Though mitochondria are estimated to have entered host cells some 2 billion years ago, the integration of mtDNA elements into the nuclear genome appears to be an ongoing process [23]. Mitochondria contain, on average, 2–10 mtDNA molecules, depending on their state within the fusion-fission cycle [24]. Because human mtDNA lacks mutation-suppressing elements such as intron sequences, it is estimated that mtDNA has a 10-fold higher accumulation of mutations than nuclear DNA [25]. Moreover, the small size of mtDNA molecules (approximately 16.5 kb in humans) increases the frequency of primary mutations in mtDNA sequence, which may result in ETC deficiencies and thus lead to impaired mitochondrial function. Mechanisms regulating the upkeep of mtDNA are therefore of paramount importance in maintaining mitochondrial fitness and preventing disease [26,27].

Damage to mtDNA is, in large part, caused by ROS generated within the mitochondrion. Considering the relatively high local concentration of ROS and the significant impact of mutations in mtDNA, this is a dangerous combination. Though the mitochondrion contains no DNA maintenance machinery of its own, it was discovered that many nuclear-encoded proteins involved in DNA maintenance and repair are also targeted to mitochondria [28]. The best characterized DNA repair mechanism in mitochondria is base excision repair, whereby a damaged (improperly paired) base is excised, the gap is filled, and the new base is ligated onto the recipient nucleotide [29]. Additionally, nuclear proteins required for nucleotide excision repair in the nucleus also localize to mitochondria upon dysfunction, hinting that this pathway may exist in mitochondria [30].

Another problem the mitochondrion must circumvent is single- or double-strand DNA breaks, caused by ROS-induced sugar damage, or by the failure of topoisomerases to relieve torsional stress following transcription. Single-strand DNA breaks recruit enzymes common to base excision repair to repair the damaged 5′ and 3′ nucleotides, along with the tyrosyl-DNA phosphodiesterase 1 (TDP1) protein, which repairs breaks originating during DNA replication, at which time most single-strand breaks occur [31]. Double-stranded breaks in nuclear DNA are extremely deleterious to the cell, and therefore the nucleus has developed three pathways to repair such breaks in nuclear DNA, including end-joining and homologous recombination [28]. Preliminary evidence has suggested that these repair pathways may also function in mitochondria [32,33]. Finally, mitochondria have the ability to repair insertion-deletion loops, arising from base-base mismatches, by a mismatch repair pathway [34].

When the mitochondrion is unable to repair mtDNA damage, the mitochondrion may segregate damaged mtDNA into a region of the mitochondrion which, following fission, will immediately decrease in respiratory activity and will be targeted for mitophagy, destroying the damaged mtDNA molecules within [16]. As an extreme and rare last alternative, excessive amounts of mutated and/or unrepaired mtDNA may trigger apoptosis of the entire cell [35]. As a functional system, mechanisms for mtDNA maintenance and repair thus play a critical role in maintaining mitochondrial fitness.

### Mitochondrial metabolism varies among tissues

Like differentiated cells, mitochondria are specialized to perform specific functions within tissues. For example, mitochondria in the liver are primarily biosynthetic, while in the heart mitochondria are primarily ATP generating. The co-evolution of mitochondria within tissues has led to the development of mechanisms for nuclear-encoded proteins to control many mitochondrial functions, greatly contributing to metabolic homeostasis in humans [36]. Though evidence strongly supports the nucleus controlling many of the mitochondrion’s activities, the mechanisms behind many putative pathways remain undefined. Some nuclear-encoded proteins, such as mitochondrial transmembrane carrier proteins for glutamate and ATP-Mg/P_i_ exchange, have been found to vary among species and tissues, often coinciding with the specialized function of that particular cell type [37]. Another identified modification is the hyper-phosphorylation of proteins comprising mitochondrial respiratory complexes in cardiac muscle cells, which stimulates ETC activity and thereby ATP production [38]. Additionally, the differential or tissue-specific expression of proteins such as transporters or gene regulatory proteins is one way in which mitochondria of different cell types can be specialized.

Within tissues, mitochondria also participate in signaling by calcium buffering. One of the most important physiological roles of mitochondria, aside from ATP production, is the storage of calcium (Ca^2+^) ions. Calcium ions enter mitochondria by a calcium ion uniporter, where they are sequestered. The speed and mechanism by which calcium ions are released from mitochondria back into the cytosol are highly variable among mitochondria of different tissue types [39]. Since very small (nanomolar) fluctuations in cytosolic calcium ion concentration have the ability to influence signaling pathways, and thereby alter the cell’s physiology, the sequestration of calcium ions within mitochondria provides an important control over intracellular signaling. As discussed above, increased mitochondrial calcium levels also stimulate ATP synthesis, and thus there is a direct link between calcium sequestration within mitochondria and mitochondrial respiratory activity at the level of the ETC.

### ER-mitochondria interactions mediate cellular processes

**Figure 2:**
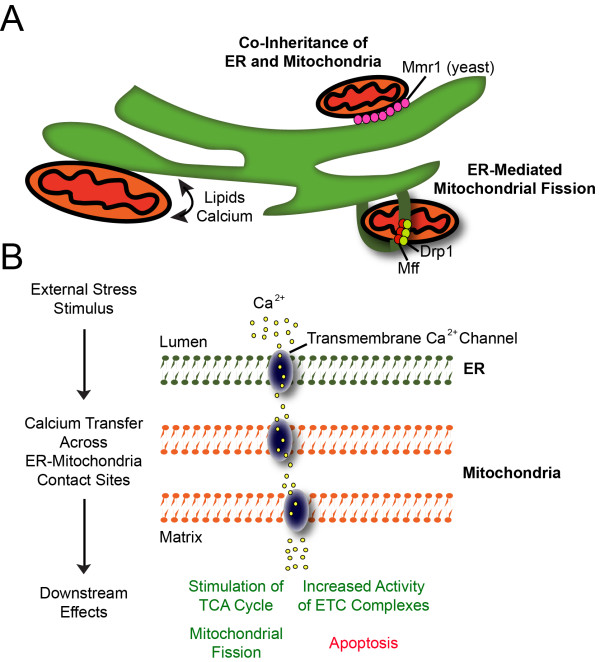
**ER and mitochondria interact intimately to perform cellular functions. (A)** ER-mitochondria contact sites mediate a variety of processes. ER-mitochondria contact sites are sites of lipid and calcium exchange, used for modifying membrane lipids and sequestering calcium ions for modulating mitochondrial function as targets of intracellular signaling cascades. Such contact sites are also thought to be regulators of mitochondrial fission in a recent hypothesis that holds that ER extensions aid in the recruitment of Mff and Drp1 to mitochondria, and furthermore provide mechanical force for fission. Additionally, recent findings show that the ER and mitochondria are inherited in a co-dependent manner in yeast, facilitated by the Mmr1 protein, which binds mitochondria to cortical ER sheets in the yeast bud tip. **(B)** In response to an external stimulus indicating cellular stress, calcium ions sequestered in the ER lumen are released into the cytosol by the transmembrane inositol 1,4,5-triphosphate receptor protein. Released calcium ions then enter ER-tethered mitochondria by an unknown transmembrane calcium channel located in the outer mitochondrial membrane, followed by translocation into the mitochondrial matrix by the mitochondrial calcium uniporter. The targets of calcium ions in the matrix primarily include energy-producing processes, but can also lead to apoptosis in cases of severe cellular stress.

Mitochondria and ER are not stand-alone, independently functioning organelles. Rather, their close interaction is crucial for a number of processes including mitochondrial fission, calcium buffering, lipid flux, and organelle inheritance (Figure 2A) [40,41]. Mitochondria and ER exchange a number of lipids, some which contribute to mitochondrial membrane elongation, and others which are chemically modified in the mitochondrion and then translocated back to the ER [42]. Dysfunctions in lipid flux are also a hallmark of several cardiovascular diseases which are also closely linked to deficits in ETC activity, and thus further identifying elements involved in ER-mitochondrial lipid exchange is of great interest [41,43].

Perhaps one of the most important functions of ER-mitochondria contact sites is the regulation of mitochondrial fission. It is thought that the ER encircles mitochondria, providing an initial constriction before the Drp1 protein provides the mechanical force to carry out fission [12,13,44]. It may also be that the ER regulates the recruitment of proteins required for fission, such as Drp1, to mitochondria from the cytosol. Research in recent years has greatly increased our understanding of the physical and functional relationship between the ER and mitochondria, and the next focus will be to further investigate the molecular mechanisms facilitating these interactions, how these interactions may be altered in mitochondrial-associated diseases.

In addition to lipid flux, ER-mitochondria contact sites are crucial for calcium buffering between the two organelles. Early studies showed that increased calcium efflux from the ER directly resulted in increased mitochondrial calcium levels, thereby establishing that ER and mitochondria can exchange calcium ions in a regulated fashion (Figure 2B) [45]. Mitochondria and ER both sequester calcium ions, which can later be released into the cytosol, thereby activating calcium-dependent signaling pathways involved in a multitude of processes [46]. As discussed above, mitochondria are not only storage organelles for calcium ions, but are also targets of calcium ions, working as signals to regulate the mitochondrion’s metabolic activity and other processes [47,48]. It is thought that a key function of calcium exchange is to activate calcium-dependent mitochondrial processes, such as the TCA cycle, made possible by the high concentration of calcium ions in the ER in contrast to the cytosol. Another likely function is that mitochondria and ER can co-regulate calcium ion release for cellular signaling, especially when a high concentration of calcium is required, such as the activation of the apoptotic cascade [49,50]. Fluctuations in calcium concentration is also an activator of mitochondrial fission at ER-mitochondria contact sites, thus necessitating a nearby calcium source [8,41].

In summary, interactions between mitochondria and the ER have been implicated in almost all mitochondrial processes, further adding to the complexity of mitochondrial function in health and in pathogenesis. These key processes, described here, are likely working in concert with one another to synergistically regulate mitochondrial and ER function. Most importantly, calcium buffering at ER-mitochondria contact sites directly influences ETC activity, and thereby influences mitochondrial respiration.

### Diet and nutrition influence mitochondrial processes

Diet and nutrition have the ability to influence mitochondrial function, and in turn the effects of mitochondrial-associated diseases. As discussed, energy sources provided by diet include carbohydrates and fats. Carbohydrates are broken down by the glycolysis pathway and TCA cycle to yield reduced NADH and FADH_2_, which are shuttled to the ETC for ATP production. Fats, on the other hand, are broken down by β-oxidation to yield reduced NADH and FADH_2_, as well as acetyl-CoA, which will enter the TCA cycle. Additionally, in the liver and kidneys, ketone bodies are produced as a byproduct of fatty acid β-oxidation. Ketone bodies are high-energy compounds, which can be transported via the blood to other tissues, where they are processed and enter the TCA cycle [6,7]. Ketone bodies thus have the ability to serve as an alternative energy source for tissues in situations of impaired glucose oxidation.

Though clinical data investigating how diet influences mitochondrial function is currently quite limited, one well studied diet associated with influencing mitochondrial function is the ketogenic diet. The ketogenic diet is a diet rich in fat and low in carbohydrates, designed to stimulate ketone body biogenesis by replacing carbohydrates with fats as a primary energy source. As discussed below, many mitochondrial pathologies arise from defects in mitochondrial metabolism. In some cases of disease, mutations affect proteins required for glucose oxidation (also causing ETC inhibition), leading to the inability of glucose to serve as a primary carbon source. In these cases, the use of a ketogenic diet in patients has been reported to be an effective therapy for treating impaired glucose oxidation, since stimulating fatty acid oxidation in mitochondria bypasses the glucose oxidation pathway and provides a glucose-independent energy source [51]. In this type of therapy, ketone bodies can serve as an energy-providing source in cases where the processing of carbohydrates becomes compromised. In one well studied example, a ketogenic diet has been successful in treating epilepsy, a symptom of mitochondrial ETC defects in hippocampal neurons [51].

Most studies investigating how diet modulates mitochondrial function have been performed in rodent, fly and cell line models harboring wild-type mtDNA, or mtDNA carrying mutations associated with known metabolic diseases. In laboratory models, ketone bodies have been shown to promote the replication and translation of wild-type mtDNA in conditions of heteroplasmy, in which a cell or individual harbors both wild-type and mutated mtDNA copies [52]. In a mouse model of a mitochondrial myopathy, it was shown that a ketogenic diet positively affects mitochondrial function and partially relieves some effects of mitochondrial myopathies [53]. More recently, some laboratory studies have focused on how diet composition in terms of protein, fat and carbohydrate amounts influence mitochondrial function. For example, in a *Drosophila* model of impaired ETC function, a high carbohydrate to protein ratio triggered the appearance of mitochondrial defects [54]. Therefore, there is some evidence that alterations in diet may have the ability to alleviate certain mitochondrial phenotypes in disease patients.

Despite a growing number of clinical and laboratory examples of diet’s effect on mitochondrial function, little is known about how this effect is brought about from a biochemical basis. However, further study into how diet influences mitochondrial function is likely to be very fruitful for developing novel therapies. As discussed below, nearly all mutations and defects associated with mitochondrial pathologies result in decreased activity of ETC complexes. Currently, in order to treat these diseases, researchers and clinicians must first identify the individual mutations and proteins involved in the pathology before attempting to design a therapy, which will only be useful for treating that specific form of disease. In contrast, diet appears to be able to directly influence mitochondrial metabolism, and therefore may have the ability to alleviate or suppress metabolic defects associated with mitochondrial pathologies. Therefore, by developing a diet regiment which increases metabolic function in cases of defects in mitochondrial function, one single diet plan may have the ability to treat a wide range of mitochondrial pathologies.

## Diseases associated with functional or genetic mitochondrial defects

### Mitochondrial myopathy

**Figure 3:**
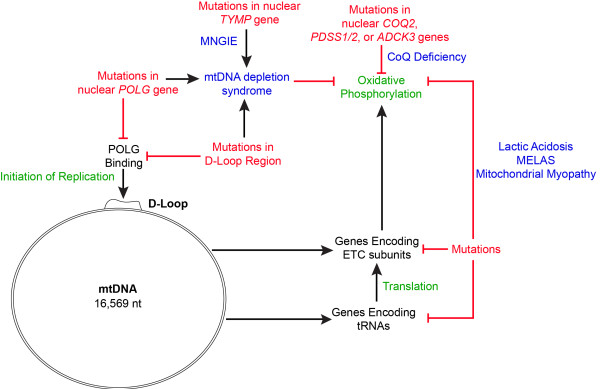
**Mutations affecting oxidative phosphorylation are the foundation of mitochondrial-associated metabolic disorders.** Mutations (red) in genes critical for mitochondrial processes (green) lead to mitochondrial-associated diseases (blue). Human mtDNA encodes 13 proteins (including subunits of ETC complexes) and 22 tRNAs on a circular genome. A D-loop region is the site of mtDNA polymerase gamma binding. Mutations within the D-loop region, or in the *POLG* gene, lead to defects in mtDNA replication and eventually mtDNA depletion. In the case of MNGIE, mutations in the nuclear *TYMP* gene, which encodes a protein involved in nucleotide synthesis, also lead to defects in mtDNA replication due to an unbalanced nucleotide pool. Mitochondria that have no mtDNA copies can no longer synthesize ETC subunits encoded by the mtDNA, leading to defects in oxidative phosphorylation. In another example of a mutation in a nuclear gene affecting mitochondrial function, mutations in genes encoding proteins required for CoQ synthesis lead to CoQ deficiency and therefore defects in electron transfer in the ETC, resulting in defects in oxidative phosphorylation. Finally, mutations in mtDNA genes themselves, including genes encoding ETC subunits or tRNAs essential for mitochondrial gene expression, lead to defects in the synthesis of ETC protein subunits or non-functional complexes. Such mutations comprise the majority of mutations associated with mitochondrial-associated diseases (excluding *POLG* mutations), and have been associated with lactic acidosis, MELAS, and mitochondrial myopathies.

Mitochondrial myopathy (also: mitochondrial encephalomyopathy) is a disease in which mitochondria within muscle fibers exhibit defects in function and dynamics, leading to weakness of the muscle and accumulation of mitochondria within the fiber, giving rise to the characteristic "ragged red" fibers seen upon Gömöri trichrome staining. Some patients experience mitochondrial myopathy as constant muscle weakness, while others only experience weakness upon more intense exercise, and in some forms is accompanied by other more intense symptoms, such as epilepsy [55]. Mitochondrial myopathies most commonly arise from mtDNA mutations leading to defects in oxidative phosphorylation, and thereby ATP production, in muscle fibers. The mtDNA mutations causing myopathies are most frequently either large-scale deletions or point mutations in genes encoding tRNAs, rRNAs, or proteins (Figure 3) [56]. In most cases, only a portion of mtDNA molecules within mitochondria carries the responsible mutations, while other copies are non-mutated [57]. Additionally, mutations in gene products associated with maintaining proper mitochondrial dynamics lead to disturbances in mitochondrial biogenesis and fusion, resulting in the accumulation of mitochondria within fibers and the "ragged red" phenotype.

Mitochondrial myopathies are difficult to detect, as clinical symptoms of mitochondrial disorders often present with many other effects, such as liver failure, stroke-like symptoms, diabetes or other symptoms. Thus, it is difficult to estimate the actual number of patients with mitochondrial myopathy, and what portion of these cases is inherited or sporadic. However, it is estimated that approximately 12.48 per 100,000 individuals are affected by mitochondrial disorders or are at risk to develop such a disorder, and a significant portion of these are expected to present symptoms of mitochondrial myopathies [58]. Some therapies to treat mitochondrial myopathies have been clinically investigated, such as the incorporation of satellite cells carrying wild-type mtDNA into myocytes during wound healing [56], or the transplantation of embryonic myocytes containing wild-type mtDNA in an attempt to decrease mutant copy number [59]. Other treatment possibilities, such as switching to a ketogenic diet to suppress the effects of myopathies, are currently being investigated [53]. Despite these attempts, there remains today no clinical treatment for mitochondrial myopathies. Because mitochondrial myopathies affect nearly every organ system, the characterization and treatment of myopathies remains a priority in treating mitochondrial-associated disorders [60]. As described below, mutations in a wide variety of genes, all of which directly or indirectly affect mitochondrial respiratory function, often lead to myopathy-like symptoms, characterized by muscle weakness and dysfunction in a wide variety of organs, such as the heart, kidneys, and intestine.

### Chronic lactic acidosis

Mutations in genes encoding mitochondrial proteins required for the assembly and function of ETC complexes, as well as the use of certain prescription drugs and other physiological stress conditions have all been associated with the onset of LA [61]. Lactic acidosis (LA) is characterized by the buildup of lactate due to decreased mitochondrial respiration, which can lead to the acidification of tissues. In healthy human cells, ATP is preferentially generated aerobically by mitochondrial respiration. However, due to a genetic or functional defect in the mitochondrial ETC, cells must switch from respiration to glycolysis in an attempt to compensate for the ATP deficit resulting from mitochondrial dysfunction. Therefore, patients have higher than normal lactate levels, which can lead to acidification of tissues. What exactly causes defects in mitochondrial function is unknown; however, the defect appears to be present upon inheritance of mitochondria from the mother, as many cases are congenital. Nonetheless, some cases are also sporadic or induced, for example in the case of patients taking metformin, a drug prescribed to treat diabetes [62]. Patients suffering from high lactate levels can suffer from tissue acidification if left untreated. The acidification of tissues can cause system-wide disturbances in physiological function, and thus it is critical that chronic LA is detected and treated. Currently available treatments include the administration of ion-containing fluids to help raise systemic pH back to near-normal levels [63]. Most patients suffering a chronic form of LA are well treated and no acidification is detectable, even though systemic lactate levels can be up to five times higher than in healthy patients. However, upon acute illness such as infection or consumption of metformin, blood pH may lower and cause symptoms including vomiting, deep and rapid breathing, and pain in the abdominal region (caused by impaired intestinal function) [64].

### MELAS

Myopathy, encephalopathy, lactic acidosis, and stroke-like episodes (MELAS) are characterized by stroke-like episodes and seizures, along with other myopathy-like symptoms such as fatigue and muscle weakness [65]. MELAS onset begins before age 40, and is progressive and fatal. In 80% of cases, patients carried a single mtDNA point mutation affecting complex I and/or tRNA function, and is thus inherited from the mother [66,67]. MELAS patients also show symptoms of LA. Like many mitochondrial-associated pathologies, it is difficult to clinically identify and there is currently no treatment available [60].

### CoQ deficiency

The mitochondrial coenzyme Q_10_ (CoQ) is an essential component of the electron transport chain, shuttling electrons between mitochondrial complexes I and II, and from electron-transferring-flavoprotein dehydrogenase (ETF-DH) to complex III, and acts as an anti-oxidant and required cofactor for pyrimidine biosynthesis [68]. Mutations in the nuclear *COQ2*, *PDSS1/2*, and *ADCK3/CABC1* genes, all of which encode enzymes that are required for CoQ biosynthesis, can lead to deficiencies in CoQ [69]. CoQ deficiency is detectable by high-performance liquid chromatographic analysis of skeletal muscle tissue, and presents symptoms such as skeletal muscle breakdown, muscle weakness, seizures, migraines, ptosis (i.e. the drooping/falling of a body part), and lactic acidosis [70]. In contrast to most other mitochondrial-associated disorders, CoQ deficiency can be treated by oral supplements of CoQ in the case of *COQ2* mutations, or riboflavin supplements in the cases of *ETFDH* mutations [69]. Patients with CoQ deficiencies due to *PDSS2, COQ9*, and some other closely associated mutations have not shown positive responses to CoQ supplements [71]. Thus, while the treatment of CoQ deficiency has had success in many cases, there are still some cases where treatments need to be identified.

### mtDNA depletion syndrome

MtDNA depletion syndrome (MDS) is characterized by the severe decrease in mtDNA levels in mitochondria of various tissues. The main gene products of mtDNA are required for proper assembly and function of the respiratory chain complexes, and thus its depletion results in severe ETC dysfunction in nearly every tissue, resulting in a severe systematic depletion of ATP. Congenital mutations manifest upon birth, when the newborn’s metabolism switches from anaerobic to aerobic [72]. Mutations in any of 10 identified genes can give rise to MDS, and it is expected that more will be identified. Most mutated genes causing MDS are involved in nucleotide synthesis, and can be identified by PCR or Southern blotting [73]. MDS is one of the more common mitochondrial-associated metabolic disorders, and has been identified as the underlying cause of mitochondrial dysfunction in 11% of children less than 2 years presenting symptoms. Most cases are fatal, and currently no treatment exists, though some symptoms are improved with CoQ treatment. Given its relative frequency, and severity of symptoms, the investigation of mutated genes giving rise to MDS, and possible treatments for it, is currently a popular field among researchers [74].

### MNGIE

Along with CoQ deficiency, MNGIE is a well-characterized mitochondrial-associated disease for which a clinical treatment has been identified. Mitochondrial neurogastrointestinal encephalomyopathy (MNGIE) is a rare mitochondrial disease, reported in about 100 cases, which arises in patients carrying a recessive mutation in the *TYMP* gene, causing a deficiency in thymidine phosphorylase [75]. Like other mitochondrial disorders, MNGIE presents a wide range of symptoms in patients, including muscle weakness, ptosis, peripheral neuropathy, gastrointestinal dysfunction, leukoencephalopathy (i.e. defects in the white matter in the brain), cachexia (i.e. loss of weight, muscle atrophy, fatigue, etc.), and often results in mtDNA depletion [69]. The disease progresses rapidly, with an onset around age 19, and often leads to fatality, with an average lifetime of 37 years when left untreated [76].

Mutations in *TYMP* lead to a deficiency of thymidine phosphorylase. Thymidine phosphorylase is required for the assembly of nucleotides, namely the processing of thymidine and deoxyuridine into thymine and uracil, respectively. The thymidine phosphorylase deficiency resulting from MNGIE leads to the accumulation of thymidine and deoxyuridine, detectable by HPLC [77]. It is believed that due to this accumulation, there is an imbalance in the nucleotide pool available in mitochondria for mtDNA replication and transcription, and this leads to mtDNA instability and degradation [78]. Research in the treatment of this disease has focused on removing the excess pools of thymidine and deoxyuridine from the blood of MNGIE patients. Therapies such as hemodialysis [79] and platelet administration [80] have shown to be effective in reducing blood plasma levels of these two nucleosides, and the subsequent alleviation of symptoms. Most recently, it has been proposed that allogenic hematopoietic stem-cell transportation (AHSCT) would be most effective in treating MNGIE, with transplanted cells of a healthy genetic background responsible for restoring the proper dNTP pool balances. Treatment for MNGIE using AHSCT is currently in planning for clinical trials [69].

### POLG mutations

As described earlier, given the critical metabolic functions of its gene products, mtDNA maintenance and replication is essential for proper mitochondrial function. It was originally thought that given the molecule’s small size and high susceptibility to loss-of-function mutations, mutations in mtDNA would underlie most mitochondrial pathologies. However, pathology arising from *POLG/POLG2* mutations is one example of a mitochondrial-associated disease caused by mutation of a nuclear gene. The replicative polymerase in mitochondria is the mtDNA polymerase γ, made of two subunits encoded by the *POLG* and *POLG2* genes. In humans, *POLG* and *POLG2* are located on nuclear chromosomes 15 and 17, respectfully. Mutations in *POLG/POLG2* can be inherited or spontaneous, and other chemical modification or inhibition of the polymerase itself (e.g. induced by anti-HIV drugs) also leads to similar symptoms [81]. To date, around 200 disease-associated *POLG/POLG2* mutations leading to ETC dysfunction have been reported and catalogued in a POLG mutation database ( http://tools.niehs.nih.gov/polg/).

Mutations in *POLG/POLG2* often lead to mtDNA depletion, with symptoms similar to MDS or MNGIE [82]. It is also documented that mutations affecting genes encoding other proteins necessary for replication (e.g. factors at the replication fork) also lead to mtDNA depletion [83]. It is estimated that 12.48 per 100,000 adults and children have or are at risk to have a develop mitochondrial disorder, and that 25% of those adults will be found to have *POLG* mutations [84]. Given that ~2% of the population carries harmful *POLG* mutations [85], and given the relatively high frequency and severity of this disease, future research into possible therapies is also receiving a great deal of attention [86].

### Mitochondrial dysfunction in type 2 diabetes

As of 2011, it is reported that 8.3% of the U.S. American population suffers from diabetes; some 90% of which have adult-onset type 2 diabetes ( http://www.diabetes.org/). The hallmark characteristic of type 2 diabetes is the elevated level of blood glucose, caused by insulin deficiency and insulin resistance [87]. The biological role of insulin, secreted by pancreatic β-cells, is to stimulate glucose uptake from the blood into target cells that require carbohydrates for energy, including in the liver and skeletal muscles. Though this disease arises from a complex set of factors including genetic predisposition and lifestyle, mitochondrial dysfunction has been identified within various aspects in the pathogenesis of this disease. A variety of molecules have been associated with contributing to insulin resistance, when target cells no longer respond to insulin signals to take up glucose from the blood, resulting in high blood glucose and energy-deprived tissues. Among these, fatty acids stand as prominent inhibitors of glucose uptake [88]. In skeletal muscle, increased levels of fatty acids in blood plasma result in increased intracellular acyl-CoA concentrations, activating an intracellular signal cascade ultimately ending with the suppression of insulin-dependent glucose transport [89]. It is believed that mitochondrial functional defects play a key role in the development of insulin resistance. First, it is likely that defects in mitochondrial β-oxidation result in increased fatty acid levels, contributing to fatty acid-induced insulin resistance [90]. The causes for defects in β-oxidation are thought to be spontaneous mtDNA mutations that accumulate upon ageing [91]. Additionally, it may be that such mutations impair the ATP-generating ability of mitochondria, which in turn leads to a deficit of ATP, which is required for the transport of glucose and insulin. One study found that mitochondria in skeletal muscle cells are smaller in number and size and exhibit reduced ETC activity in patients with type 2 diabetes, supporting the hypothesis of inherited mitochondrial defects in type 2 diabetes [92].

While impaired mitochondrial β-oxidation leading to insulin resistance is perhaps the most well supported example of mitochondrial involvement in type 2 diabetes, a variety of other hypotheses exist. For example, some studies support that pancreatic β-cell dysfunction (i.e. impaired insulin production) contributes more to pathogenesis than insulin resistance [93]. This is supported by a diabetes-associated mtDNA mutation in a gene encoding a tRNA, leading to reduced insulin secretion [94]. Additionally, mitochondria in type 2 diabetes are suspected to be involved in numerous aspects of disease pathogenesis as well as responsible for some downstream effects after onset [95,96]. Importantly, at the core of mitochondrial involvement in type 2 diabetes, as well the majority of all mitochondrial-associated metabolic diseases, is defects in ETC activity or fatty acid oxidation. Therefore, ongoing research into therapies for other mitochondrial-associated diseases is also likely to have implications for treating certain aspects of type 2 diabetes, and vice versa.

### Altered mitochondrial function in cancer

In addition to strictly metabolic disorders, mitochondrial activity is affected in cancer patients. Often, tumor progression is associated with increased mitochondrial respiration (due to rapidly growing cells) and therefore increased ROS production. With increased ROS production comes an increased risk of mtDNA mutation. MtDNA mutations are thus a hallmark of many cancer patients. A large portion of mutations occurs in the noncoding D-loop region of the mtDNA, which is responsible for the initiation of replication. It appears that mtDNA mutations may not only be consequences of tumorigenesis, but may aid in the process, evidenced by a high correlation between stage of progression and specific mtDNA mutations [97].

Since the D-loop region is required for polymerase binding and regulation of initiation of replication, it is thought that these mutations frequently inhibit mtDNA replication [97]. Indeed, it has been documented that low mtDNA copy number is a characteristic feature of numerous cancers including renal cell carcinoma [98], lung cancer [99,100], and breast cancer [101]. The correlation between low mtDNA copy number and how that may benefit cancer cell survival remains to be elucidated. More recent research into mitochondria in cancer cells has identified other variations in mitochondrial function, such as the inhibition of mitochondrial calcium uptake to promote cancer cell survival [102]. Recently it was reported that mitochondrial miRNAs may also play a role in modulating mitochondrial function and energetic in cancer situations, in which miRNA regulation is altered [103,104]. Further research into mitochondrial variation in tumor progression will likely provide the clinical community with new biomarkers for following tumorigenesis as well as novel drug targets.

### The role of mitochondrial dysfunction in heart failure

In a mechanism similar to mitochondrial dysfunction in cancer, mitochondrial dysfunction has also been implicated in heart failure [105]. The role of mitochondria in cardiac muscle is paramount due to its function as an organ of high-energy demand. Previously, studies on patients with chronic heart disease revealed decreased ETC complex activity, and identified a non-pathological point mutation in the mtDNA-encoded cytochrome *b* gene [106,107]. It is believed that a key event in the onset of heart failure is impaired blood flow both to and within the heart. Impaired heart flow leads to reduced oxygen availability at the ETC of cardiac mitochondria; since oxygen is the final electron acceptor in the ETC, a deficit of oxygen leads to the accumulation of electrons at ETC complexes. Accumulation of electrons at ETC complexes induces ROS production, which serves as a stress response to signal that oxygen availability is low [108]. ROS production, however, leads to mtDNA damage and mutations. Accordingly, mtDNA mutations are frequently observed in heart failure, leading to impaired ETC activity, and in some cases mtDNA loss and loss of mitochondrial mass [109].

Mitochondrial dysfunction caused by the accumulation of ROS is characteristic of heart failure and other mitochondrial diseases. As such, it has been proposed that mitochondrial dysfunction can be alleviated by administering patients antioxidant compounds, which act as ROS scavengers and thereby reduce their harmful effects and reduce the frequency of mtDNA mutations [110]. Only one clinical trial with antioxidants has been carried out, with the goal of alleviating mitochondrial defects in Parkinson’s disease [17,110]. The trial was unsuccessful, largely due to the difficulty in targeting the compound to mitochondria. Nonetheless, recent studies in animals [111] and cell culture models [112] have successfully delivered antioxidant compounds or enzymes to mitochondria, and observed a decrease in ROS-associated defects. Thus, the use of antioxidants seems to be a promising avenue for treating ROS-induced mitochondrial dysfunction, but with much work still ahead before human therapies will be available.

While the role of mitochondria in heart failure is more downstream than in other metabolic diseases discussed here, mitochondrial dysfunction comprises a key step in the final stages of heart failure. Therapies targeting mitochondrial biogenesis and oxidative stress to treat heart disease have made significant advances in comparison to other mitochondrial-associated diseases, with mitochondria standing as promising targets for treatment [113].

## Conclusion

By classical definition, the core function of the mitochondrion is to act as the metabolic hub of the cell. Indeed, as we have discussed here, when mitochondrial respiratory activity becomes impaired, symptoms are visible on a systemic level. However, recent research has found that mitochondria are involved in many other additional processes, which also have the ability to influence mitochondrial function and dynamics and therefore may give rise to disease. New treatments and therapies for mitochondrial-associated diseases are now under development; and while much work remains, the identification of new executors of metabolic disease has renewed attention in developing treatments for many of these disorders [114,115]. The next years of research into further understanding mitochondrial dynamics and non-canonical roles are sure to lead to new connections between defects in mitochondria and defects in other cellular processes, and provide new possibilities for developing therapies.

## Competing interests

The author declares that he has no competing interests.
